# Olopatadine in Seasonal Allergic Conjunctivitis: Efficacy, Ocular Surface Safety, and Practical Considerations for Preservative-Free Multidose Therapy

**DOI:** 10.3390/jcm15207963

**Published:** 2026-10-15

**Authors:** Giorgio Ciprandi

**Affiliations:** Department of Medicine and Health Science, University of Molise, 86100 Campobasso, Italy; gio.cip@libero.it

**Keywords:** seasonal allergic conjunctivitis, olopatadine, benzalkonium chloride, preservative-free, ocular surface, treatment adherence, polysensitization, pollen season, antihistamine, mast cell stabilizer

## Abstract

**Background:** Seasonal allergic conjunctivitis (SAC) is the most prevalent form of ocular allergy, characterized by recurrent episodes of IgE-mediated conjunctival inflammation triggered by seasonal aeroallergens (pollens). Its clinical course typically spans 4–12 weeks for individual pollen taxa and may extend considerably longer in polyallergic patients. Olopatadine hydrochloride ophthalmic solution, a selective H1-receptor antagonist with mast cell-stabilizing properties, is one of the most extensively studied topical antihistamines for SAC and is highly ranked in comparative evidence networks. Two clinically relevant aspects have received insufficient attention: the impact of benzalkonium chloride (BAK), a preservative, on the conjunctival surface, and the implications of bottle size for treatment adherence during SAC’s prolonged symptomatic periods. **Methods:** A non-systematic narrative review of the literature was conducted. PubMed was searched through 6 October 2026 using combinations of terms addressing seasonal and allergic conjunctivitis, olopatadine, benzalkonium chloride, preservative-free formulations, ocular surface, pollen season, polyallergy/polysensitization, adherence/compliance, multidose systems, contamination, and in-use shelf life. Randomized controlled trials, comparative clinical studies, systematic reviews, meta-analyses, and clinically relevant mechanistic, toxicological, and microbiological studies were prioritized; reference lists of key articles were also screened. No formal systematic-review or PRISMA selection process was applied. Because direct comparative evidence for preservative-free versus BAK-preserved olopatadine and for bottle-size effects on adherence is lacking, evidence from related ophthalmic formulations was considered indirect and interpreted accordingly. **Results:** In the most recent network meta-analysis, olopatadine 0.1% was highly ranked among topical antihistamines for SAC (SUCRA 0.88) and perennial allergic conjunctivitis (PAC; SUCRA 0.85). However, this ranking should be interpreted together with pairwise comparisons and study-design heterogeneity, including CAC-based evidence in which alcaftadine has shown greater reduction of ocular itch at early post-challenge time points. Olopatadine acts primarily at the conjunctival surface, with low systemic exposure after topical administration. Current evidence does not establish that BAK-mediated enhancement of intraocular drug delivery is required; however, in conventional multidose bottles, it serves an important antimicrobial-preservative function, and any preservative-free multidose presentation must rely on validated container technology and an adequate in-use shelf life. For olopatadine 0.1% BID, a larger bottle may reduce the number of dispensing events during prolonged symptomatic periods, but a direct adherence benefit has not been demonstrated. **Conclusions:** Olopatadine remains an established topical treatment for SAC. Preservative-free formulations may avoid preservative-related ocular-surface exposure, but direct head-to-head evidence demonstrating superior ocular-surface outcomes for preservative-free versus BAK-preserved olopatadine is currently unavailable. Likewise, a larger bottle may be practically useful for BID regimens when its validated in-use period permits prolonged use, although bottle size alone should not be considered a proven determinant of adherence. Formulation choice should therefore integrate efficacy, dosing frequency, ocular-surface tolerability, container technology, in-use shelf life, cost, and the expected duration of allergen exposure.

## 1. Introduction

Seasonal allergic conjunctivitis (SAC) is an IgE-mediated type 1 hypersensitivity reaction of the conjunctival mucosa triggered by seasonal aeroallergens, predominantly pollens. It represents the most prevalent form of ocular allergy, affecting 15–40% of the general population, with epidemiological patterns influenced by environmental and climatic factors [1,2]. The clinical spectrum of allergic conjunctivitis encompasses SAC, perennial allergic conjunctivitis (PAC), vernal keratoconjunctivitis (VKC), and atopic keratoconjunctivitis (AKC), each differing in pathophysiology, severity, and chronicity [3]. The hallmark symptom of SAC is ocular pruritus, which is present in more than 75% of patients, and it is accompanied by conjunctival hyperemia, tearing, chemosis, a foreign body sensation, and eyelid swelling [4]. These symptoms significantly impair quality of life and impose a substantial socioeconomic burden [5,6]. Symptoms are primarily driven by the early-phase allergic response, mediated by histamine released from conjunctival mast cells following allergen-IgE cross-linking [7], acting through H1 and H2 receptors on neuronal fibers, vascular endothelial cells, goblet cells, and epithelial cells [8,9]. Among available topical agents, olopatadine hydrochloride is among the most extensively studied treatments for SAC. It combines selective H1-receptor antagonism with mast cell-stabilizing and anti-inflammatory properties [10,11], providing both rapid symptomatic relief and sustained anti-allergic protection [12,13,14]. The clinical relevance of this sustained efficacy has been demonstrated in 6-week environmental trials by Katelaris et al. [15] and Ciprandi et al. [16], confirming that olopatadine maintained its therapeutic effect during the study periods. A clinically important feature of SAC is polyallergy, defined here as clinically relevant allergy to multiple distinct pollen allergens, and distinguished from polysensitization, which denotes sensitization without necessarily implying clinical symptoms [17]. Climate change can alter the timing and duration of pollen exposure [18,19]. In genuinely polyallergic patients, sequential clinically relevant pollen seasons may prolong the symptomatic period, although the extent of overlap and the proportion of SAC patients requiring continuous treatment for several months vary geographically and between individuals. Two practical issues therefore merit consideration. First, BAK can exert ocular-surface toxicity [20]; however, its antimicrobial-preservative role in conventional multidose bottles must be balanced against this potential toxicity. Second, for olopatadine 0.1% BID, a 5 mL bottle may not cover a prolonged symptomatic period under commonly used drop-volume assumptions. A larger bottle could reduce dispensing frequency, but any such advantage depends on the validated in-use shelf life of the multidose system and has not been shown directly to improve adherence. This review critically appraises the evidence for olopatadine in SAC, focusing on efficacy across the pollen season, ocular-surface safety of preservative-free versus BAK-preserved formulations, and practical considerations related to treatment duration and drug supply. This is a narrative, not systematic, review. PubMed was searched through 6 October 2026 using combinations of the terms “seasonal allergic conjunctivitis”, “allergic conjunctivitis”, “olopatadine”, “benzalkonium chloride”, “preservative-free”, “ocular surface”, “pollen season”, “polyallergy”, “polysensitization”, “adherence”, “compliance”, “multidose”, “contamination”, and “in-use shelf life”. Randomized controlled trials, comparative clinical studies, systematic reviews, meta-analyses, and clinically relevant mechanistic, toxicological, or microbiological studies were prioritized; reference lists of key articles were also screened. Selection was based on relevance to efficacy, ocular-surface safety, treatment duration, preservative exposure, and formulation-related practical issues; no formal systematic-review or PRISMA selection process was applied. Because no direct trial was identified that evaluated preservative-free versus BAK-preserved olopatadine or the effect of olopatadine bottle size on adherence, evidence addressing these questions is necessarily indirect and is identified as such throughout the review.

**Figure 1:**
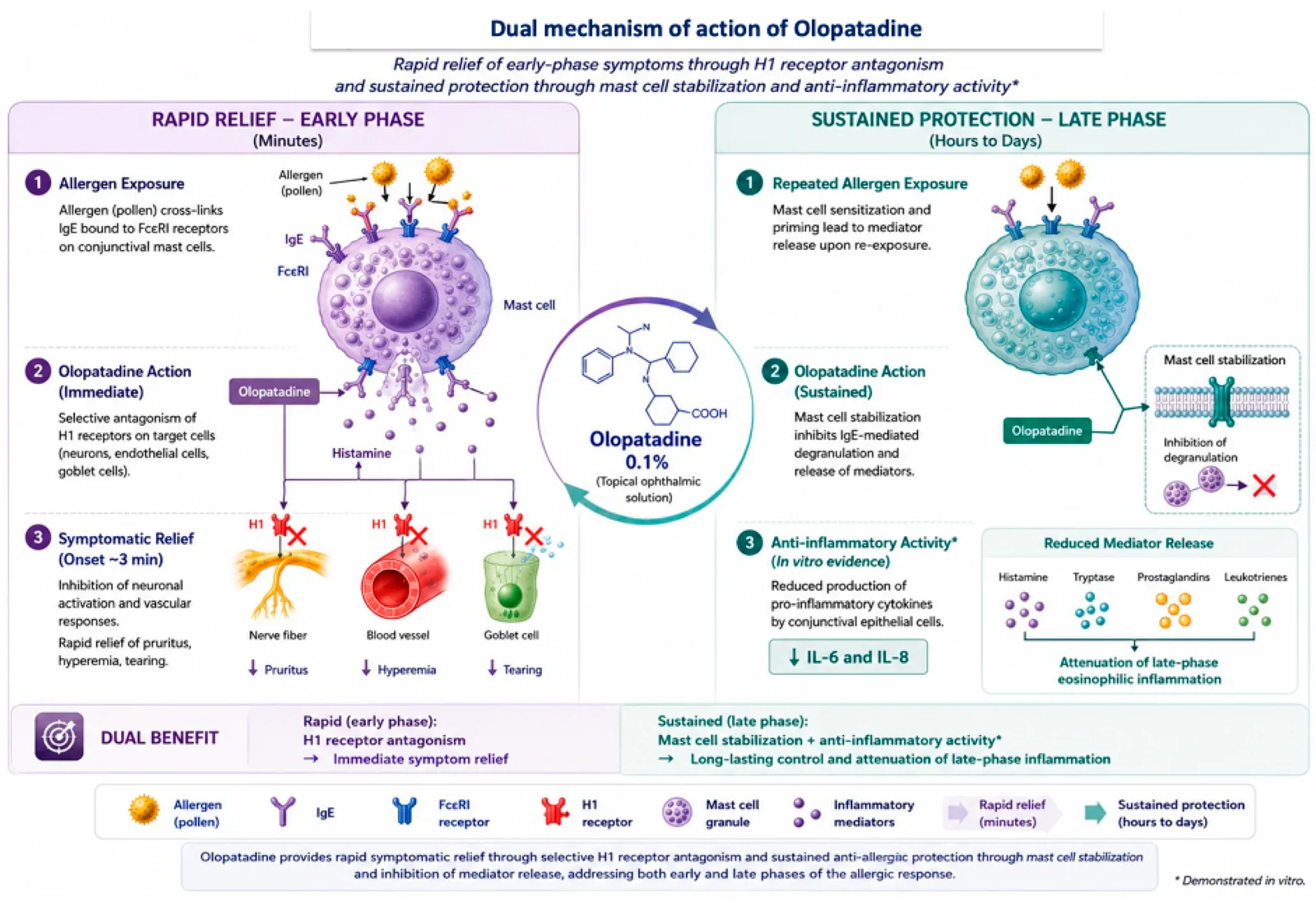
Dual mechanism of action of olopatadine hydrochloride ophthalmic solution in allergic conjunctivitis. Olopatadine combines rapid H1 receptor antagonism (early phase) with mast cell stabilization (late phase), providing both immediate symptomatic relief and sustained anti-allergic protection. IL, interleukin.

**Figure 2:**
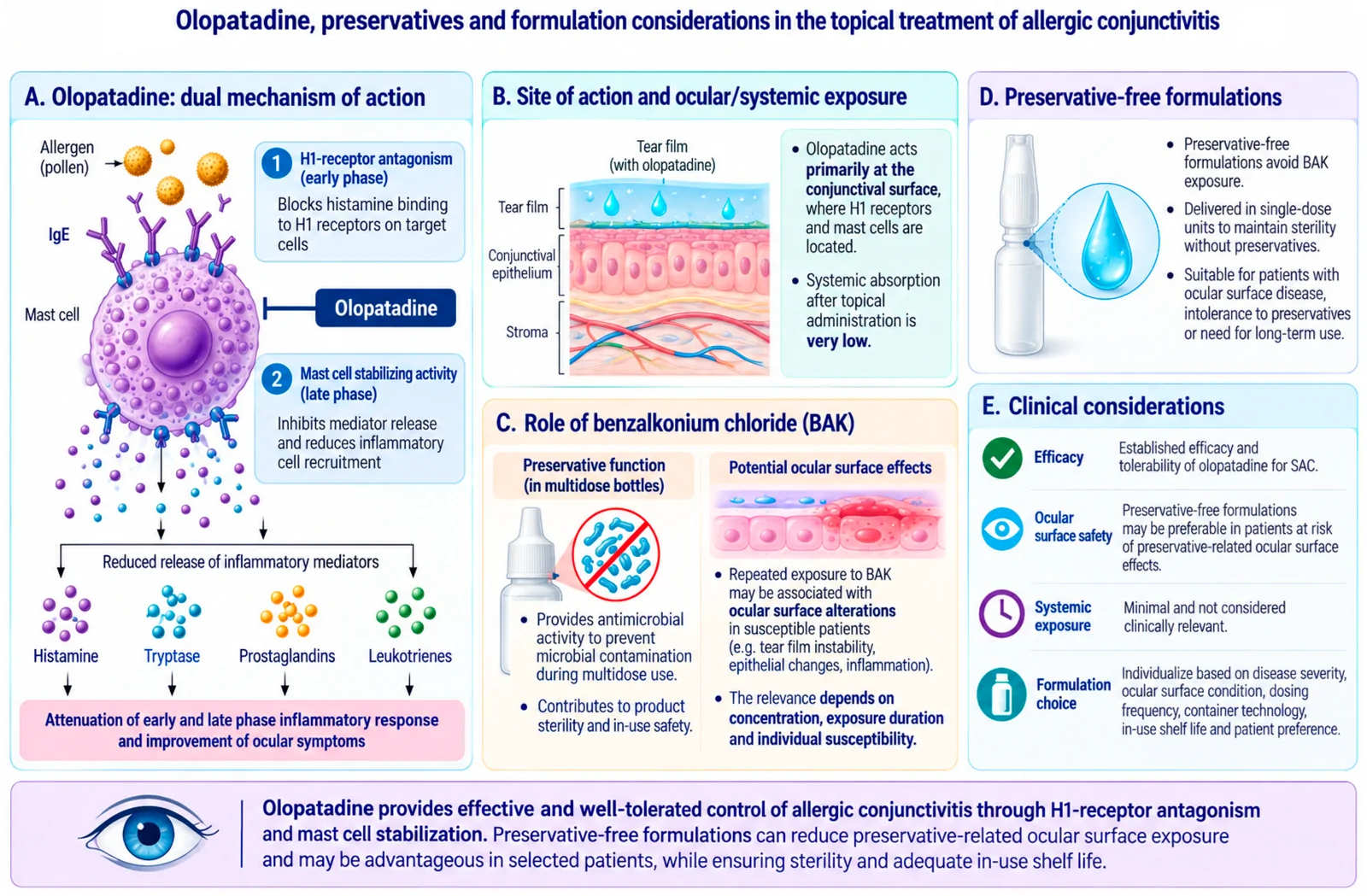
Pharmacological rationale and trade-offs relevant to preservative-free olopatadine formulations. Olopatadine has clinically relevant targets at the conjunctival surface, and current evidence does not establish that BAK-mediated enhancement of intraocular penetration is required for therapeutic efficacy. BAK nevertheless provides antimicrobial preservation in conventional multidose bottles; Preservative-free multidose formulations require validated container/closure technology and microbiological protection throughout the labelled in-use period. BAK, benzalkonium chloride.

Use of generative AI in manuscript preparation: Generative AI (OpenAI ChatGPT; web-based application, chatgpt.com, accessed 11 October 2026; exact model version not recorded) was used to assist with the preparation and refinement of scientific illustrations, including Figure 1 and Figure 2, and editorial language refinement. The author reviewed and edited the resulting material, checked it against the cited literature, and assumes full responsibility for its accuracy and content.

## 2. Pharmacology of Olopatadine: Mechanism of Action and Pharmacokinetics

### 2.1. Mechanism of Action

Olopatadine hydrochloride [(Z)-11-[3-(dimethylamino)propylidene]-6,11-dihydrodibenz[b,e]oxepin-2-acetic acid hydrochloride] is a dibenzoxepine derivative with a dual mechanism of action [21]. It selectively antagonizes histamine H1 receptors, which are key mediators of the early-phase conjunctival allergic response [8], with minimal affinity for adrenergic, muscarinic, serotonergic, or dopaminergic receptors, with a receptor selectivity profile distinct from that of first-generation antihistamines [9]. Additionally, olopatadine inhibits IgE-mediated mast cell degranulation, reducing the release of histamine, tryptase, prostaglandins, and leukotrienes [12], and suppresses pro-inflammatory cytokine production by conjunctival epithelial cells [22]. This dual mechanism provides rapid symptomatic relief through immediate H1 blockade and sustained anti-allergic protection through mast cell stabilization, with attenuation of the late-phase eosinophilic response. The mechanism of action is schematically illustrated in Figure 1.

### 2.2. Pharmacokinetics and Ocular Surface Confinement

Olopatadine is intended to act locally at the ocular surface following topical instillation. In vivo studies showed that olopatadine 0.1% applied to one eye had no detectable effect on the contralateral eye, and plasma concentrations following ocular instillation are clinically negligible [13]. Its clinically relevant targets include H1 receptors and mast cells in the conjunctival epithelium and substantia propria. Although BAK can enhance corneal permeability for some compounds, the available evidence does not establish that BAK-mediated enhancement of intraocular penetration is required for the therapeutic effect of topical olopatadine [20]. This does not diminish the distinct formulation role of BAK: in conventional multidose bottles, its principal function is antimicrobial preservation after opening. The clinical issue is therefore a balance between preservative-related ocular-surface exposure and microbiological safety, which preservative-free multidose systems must address through validated container technology and in-use stability. Available formulations include the 0.1% solution (BID), the 0.2% solution (once daily), and the 0.77% solution (once daily, approved in the U.S.), which demonstrated superiority over both lower concentrations in reducing ocular itching at 24 h in a phase 3 RCT [23]. The 0.1% formulation remains the most widely studied concentration.

## 3. Clinical Efficacy of Olopatadine in Allergic Conjunctivitis

### 3.1. Efficacy Versus Placebo

The efficacy of olopatadine versus placebo is established across a large body of RCTs using both the conjunctival allergen challenge (CAC) model and environmental designs. A systematic review and meta-analysis by Kam et al. (23 RCTs, 3388 eyes) demonstrated a pooled mean difference (MD) in ocular itch of −1.33 (95% CI: −1.43 to −1.23; *p* < 0.00001) and in conjunctival hyperemia of −0.92 (95% CI: −1.19 to −0.65; *p* < 0.00001) versus placebo [24], consistent across all subgroup analyses. In the CAC model, olopatadine 0.1% shows onset within 3 min and duration of at least 8 h [23,25,26,27,28]. Environmental trials, including the 6-week studies discussed below, provide complementary evidence under natural pollen exposure [15,16].

### 3.2. Comparative Efficacy Against Other Topical Antihistamines

Comparative data are summarized in Table 1. A network meta-analysis (28 RCTs, SUCRA methodology) ranked olopatadine 0.1% as the most effective topical treatment for both SAC (SUCRA 0.88) and PAC (SUCRA 0.85) [1]. SUCRA rankings are probabilistic summaries of a network and should not be interpreted as proof of superiority in every direct comparison or as establishing universal first-line status; their interpretation is also influenced by the designs of the contributing trials, including CAC studies. Against ketotifen, the Cochrane review found a statistically significant advantage for olopatadine at 14 days (MD −0.32; 95% CI: −0.59 to −0.06; *p* = 0.02; I^2^ = 83%) [10], while Kam et al. reported comparable overall efficacy (pooled MD −0.22; *p* = 0.31) [24], with individual environmental trials tending to favor olopatadine during peak pollen periods. Avunduk et al. found no significant difference over 30 days [29]. Against epinastine, Fukushima and Ebihara demonstrated olopatadine’s superiority in a CAC-based RCT (ocular itch at 7 min: *p* = 0.0462; conjunctival hyperemia at 20 min: *p* = 0.0273) [30], consistent with earlier findings by Lanier et al. [25]; the meta-analysis by Kam et al. showed a non-significant trend favoring olopatadine (pooled MD −0.23; *p* = 0.08) [24]. Against alcaftadine, a recent meta-analysis found alcaftadine 0.25% superior to olopatadine 0.2% in CAC-based studies (pooled MD +0.39; *p* < 0.00001; I^2^ = 0%) [31], while real-world environmental studies by Dudeja et al. [32] and Ayyappanavar et al. [33] found comparable efficacy between olopatadine, alcaftadine, and bepotastine over 1–2 weeks. These apparently divergent findings should be presented without privileging one study design: CAC studies offer standardized assessment of onset and duration, whereas environmental studies assess effectiveness during natural exposure. Together, they indicate that comparative efficacy may depend on endpoint, timing, concentration, and study setting.

Six-week environmental trials by Katelaris et al. [15] and Ciprandi et al. [16] showed that olopatadine retained efficacy during periods of higher natural pollen exposure and, in selected comparisons, its relative advantage over comparators increased as pollen counts rose. These findings support the clinical relevance of efficacy during peak exposure, but they do not by themselves establish that uninterrupted treatment throughout an entire pollen season is necessary for every patient.

## 4. Benzalkonium Chloride in Ophthalmic Formulations: Toxicological Profile and Clinical Implications

### 4.1. Mechanisms of BAK-Induced Ocular Surface Toxicity

Benzalkonium chloride (BAK) is a quaternary ammonium compound widely used as an ophthalmic preservative. However, extensive experimental and clinical evidence has established potential cytotoxic effects on the ocular surface, affecting the corneal epithelium, conjunctival epithelium, goblet cells, trabecular meshwork, and ciliary epithelium [20,34]. At the cellular level, BAK disrupts cell membrane lipid bilayers, causing increased permeability, mitochondrial dysfunction, and apoptosis. At the tissue level, it reduces conjunctival goblet cell density—critical for the integrity of the tear film mucin layer—thereby impairing tear film stability, promoting desiccation, and creating a pro-inflammatory microenvironment that may exacerbate allergic disease [34]. BAK also promotes lymphocyte infiltration, upregulates HLA-DR and ICAM-1, and induces subconjunctival fibrosis via TGF-β1/Smad3 signaling [20,34,35]. These effects may not be limited to chronic exposure: histopathological changes in the conjunctival epithelium are detectable after as few as 7 days at concentrations as low as 0.001% [20], initially asymptomatic but potentially progressive.

### 4.2. Clinical Evidence of BAK-Induced Conjunctival Damage in Anti-Allergic Therapy

Direct clinical evidence relevant to preservative exposure comes from a prospective multicenter randomized controlled trial by Marini et al. that compared bepotastine besilate 1.5% preservative-free (BB-PF) with olopatadine 0.2% BAK-preserved (OL-BAK) in 97 patients over 60 days [34]. Conjunctival impression cytology (Nelson classification) at baseline, 30, and 60 days showed that OL-BAK patients had a 27.4% decrease in normal conjunctival epithelium, whereas BB-PF patients showed a 20.5% increase. The probability of normal epithelium was 2.7 times greater in the BB-PF group (OR = 2.72; 95% CI: 1.50–4.94; *p* < 0.001) [34], with three cases of epithelial metaplasia in the OL-BAK group versus none in BB-PF. Because this study compared different active molecules as well as different preservative status, the observed difference cannot be attributed to BAK alone and does not constitute a direct comparison of preservative-free versus preserved olopatadine. Moreover, the 60-day exposure period is most relevant to patients treated continuously for prolonged periods and should not be extrapolated uncritically to shorter or intermittent SAC treatment. Nevertheless, the findings are clinically relevant because allergic conjunctivitis itself is associated with conjunctival inflammation and epithelial disruption, making ocular-surface tolerability an important consideration during repeated or prolonged topical therapy.

### 4.3. Balancing the Antimicrobial Role of BAK and Ocular-Surface Considerations

BAK has an established antimicrobial-preservative role in conventional multidose ophthalmic formulations and can also enhance corneal permeability. For olopatadine, whose clinically relevant targets include the conjunctival surface, the available evidence does not demonstrate that BAK-mediated enhancement of intraocular penetration is required for therapeutic efficacy [12,13]. Thus, the rationale for considering preservative-free olopatadine is not that BAK has ‘no value’, but that its microbiological function must be weighed against potential ocular-surface toxicity. Preservative-free multidose products can be clinically appropriate only when the container/closure system itself provides validated protection against microbial ingress throughout the labelled in-use period. Studies of preservative-free multidose ophthalmic preparations show that contamination can occur when microbiological protection is inadequate [36,37], whereas dedicated multidose container technology can maintain microbiological integrity without a conventional preservative [38]. Accordingly, bottle volume alone cannot determine the duration of safe use: the labelled in-use shelf life and the specific dispensing technology are decisive. These pharmacological and formulation considerations are summarized in Figure 2.

## 5. Duration of Seasonal Allergic Conjunctivitis and Practical Implications for Drug Supply

### 5.1. Natural History and Duration of Seasonal Allergic Conjunctivitis

SAC is a condition whose clinical course is determined by the temporal pattern of environmental allergen exposure [39]. The seasonal estimates discussed here refer primarily to temperate European climates and should not be generalized to other geographic regions. In temperate Europe, pollen seasons vary by taxon: grass pollens (Poaceae), the most common SAC trigger, are airborne April–September with a 6–10 week peak [18,19]; Betulaceae and Cupressaceae have 4–6 and 3–5 week peaks respectively, from January–May [18,19]; Ambrosia peaks 4–8 weeks from July–October [18,19]; Parietaria, particularly problematic in Mediterranean regions, peaks 8–12 weeks from March–October [18,19]. Patients with true polyallergy to multiple clinically relevant pollen taxa may experience sequential symptomatic periods extending beyond a single pollen season; however, sensitization to multiple taxa does not necessarily imply clinical polyallergy, and the prevalence and degree of seasonal overlap vary substantially [17]. Therefore, prolonged uninterrupted treatment should be individualized according to symptoms and actual allergen exposure rather than assumed for all polysensitized patients. The major pollen taxa and their seasonal durations are summarized in Table 2. The 2015 Cochrane systematic review (30 RCTs, 4344 participants) found treatment durations in clinical trials ranging from 1–8 weeks, most lasting 2–6 weeks [10].

### 5.2. Bottle Size, Drug Supply, and Adherence Considerations

For olopatadine 0.1% BID (one drop per eye twice daily), drug supply depends on bottle fill volume, drop size, device characteristics, and actual patient use. Using the illustrative assumption of approximately 100–120 drops per 5 mL bottle and twice that number per 10 mL bottle, a 5 mL bottle would provide roughly 25–30 days and a 10 mL bottle roughly 50–60 days of bilateral BID dosing. These calculations are estimates rather than adherence data and should not be generalized across dispensing systems without product-specific information. A larger bottle could reduce the number of dispensing events during prolonged treatment, but no study identified in this review has directly demonstrated that bottle size improves adherence or clinical outcomes in SAC. Adherence is multifactorial and is also influenced by education, symptom burden, dosing frequency, cost, access, and tolerability. Importantly, the usable duration of any multidose bottle is limited by its labelled in-use shelf life after opening. A nominal 50–60-day drug supply is clinically meaningful only if the specific preservative-free container/closure system is validated for use over that period; otherwise, a larger fill volume could lead to wastage rather than improved continuity. The supply issue is also regimen-specific: once-daily olopatadine formulations consume approximately half as many drops as the 0.1% BID regimen and may therefore provide longer coverage from the same fill volume, subject again to labelled in-use shelf life. Consequently, a 10 mL BID bottle should not be considered intrinsically preferable to once-daily alternatives. Bottle volume, dosing frequency, in-use stability, cost, and expected treatment duration should be considered together. Table 2 provides only illustrative BID calculations, not prescribing recommendations.

## 6. Safety and Tolerability of Olopatadine

Olopatadine has shown a generally favorable safety profile across clinical trials. No serious adverse events were reported across the 23 RCTs included in the Kam et al. meta-analysis [24] or across 30 RCTs in the Cochrane review [10]. The most common adverse events—headache, mild burning or stinging upon instillation, dry eye sensation, and foreign body sensation—are self-limiting and rarely lead to discontinuation [14]. Comparative tolerability data generally favor olopatadine over ketotifen. Artal et al. found that 100% of subjects preferred olopatadine 0.1% to ketotifen 0.05% for instillation comfort, with 98% of ketotifen-treated subjects reporting burning, compared with 0% with olopatadine [40]. Leonardi and Zafirakis reported that 81% of patients preferred olopatadine 0.1% over ketotifen 0.025% for both comfort and efficacy [41], and Sarker et al. found no drug-related adverse events with olopatadine, compared with 30% stinging with ketotifen [42]. Mortemousque et al. found a trend toward better tolerability with preservative-free ketotifen 0.025% versus BAK-preserved olopatadine 0.1% at day 7 (*p* = 0.054), with 13.5% of olopatadine-treated patients experiencing instillation-related symptoms [43]. Because both the active molecule and preservative status differed between groups, this study cannot determine whether the tolerability difference was attributable to BAK, to the active drug, or to both. In pediatric SAC, Katelaris et al. found that children aged <11 years receiving olopatadine had redness scores 2 times lower than those receiving cromolyn (*p* = 0.002), with better local tolerability [15]. Ciprandi et al. confirmed good tolerability in children aged 4–11 years over 6 weeks, with only one mild treatment-related adverse event [16]. The former U.S. FDA pregnancy letter categories, including ‘Category C’, are no longer used under the Pregnancy and Lactation Labeling Rule. Available human pregnancy data are limited; therefore, use during pregnancy should be based on an individualized assessment of expected maternal benefit and potential fetal risk. Because systemic exposure after topical ocular administration is low, nasolacrimal occlusion can be used to further minimize systemic absorption.

## 7. Discussion

This review examined three clinically relevant dimensions of olopatadine use in SAC: comparative efficacy across the pollen season, ocular-surface safety of preservative-free versus BAK-preserved formulations, and practical implications of bottle size and dosing frequency for drug supply during prolonged symptomatic periods. Olopatadine 0.1% is highly ranked among topical antihistamines for SAC and PAC (SUCRA 0.88 and 0.85, respectively) [1], with a dual mechanism that provides both immediate and sustained anti-allergic protection. However, this ranking should be interpreted together with pairwise evidence and study-design heterogeneity. CAC-based comparisons have favored alcaftadine for early post-challenge itch reduction [31], whereas environmental studies have shown sustained efficacy or advantages for olopatadine against several comparators [15,16,32,33]. These findings are complementary and should be interpreted according to their respective experimental settings. The environmental trials by Katelaris et al. [15] and Ciprandi et al. [16] demonstrated sustained efficacy during pollen exposure and showed that olopatadine’s therapeutic advantage over comparators may become more evident as pollen concentrations rise. This supports ensuring effective treatment during clinically relevant exposure, but does not establish that uninterrupted treatment is required in every patient. The argument for preservative-free olopatadine rests not on any concern about efficacy, but on the potential ocular-surface effects of repeated preservative exposure. BAK has well-documented ocular-surface toxicity [20,35], but it also provides antimicrobial preservation in conventional multidose bottles. Because olopatadine has clinically relevant targets at the conjunctival surface, current evidence does not establish that BAK-mediated enhancement of intraocular penetration is required for efficacy; the relevant issue is therefore the balance between microbiological protection and preservative exposure. The histopathological findings from Marini et al. [34] are relevant: after 60 days, BAK-preserved olopatadine 0.2% was associated with a 27.4% decrease in normal conjunctival epithelium, whereas the preservative-free bepotastine group showed an increase. Nevertheless, the study compared different active molecules as well as different preservative status, so the differences cannot be attributed to BAK alone and do not constitute direct evidence of superiority of preservative-free olopatadine. Similarly, chronic glaucoma data support biological plausibility but cannot be quantitatively extrapolated to the shorter and variable exposure typical of SAC. The bottle-size argument is grounded in the practical relationship between treatment duration and drug supply. For bilateral olopatadine 0.1% BID, a 5 mL bottle may provide approximately 25–30 days and a 10 mL bottle approximately 50–60 days under the illustrative assumptions used here. A larger fill volume could therefore reduce dispensing events during a prolonged symptomatic period, but no study has demonstrated that bottle size itself improves adherence or clinical outcomes. For patients with clinically relevant allergy to multiple pollen taxa, sequential exposure may extend the symptomatic period beyond that associated with a single pollen season. However, polysensitization is not synonymous with polyallergy, seasonal overlap varies among patients and regions, and treatment duration should be individualized according to symptoms and actual exposure. The practical value of a larger preservative-free bottle also depends on its labelled in-use shelf life and dispensing technology. A preservative-free multidose system intended for prolonged use must have validated microbiological integrity throughout that period; otherwise, theoretical drug supply may exceed safe use and increase wastage. Moreover, the supply calculation applies specifically to the 0.1% BID regimen. Once-daily formulations require fewer instillations and may provide comparable treatment duration from a smaller bottle. Thus, volume should be considered together with dosing frequency, in-use shelf life, device technology, cost, access, and wastage. It is also worth noting that the tolerability advantage of olopatadine over ketotifen, consistently demonstrated in comparative studies [40,41,42], may acquire additional clinical significance when treatment is maintained for several weeks. Instillation comfort can influence willingness to continue topical therapy. However, the preservative-free ketotifen versus BAK-preserved olopatadine study [43] cannot determine whether differences in comfort were caused by the preservative because both the active molecule and preservative status differed. Finally, the pollen-count correlation data from Katelaris et al. [15] provide a useful pharmacodynamic argument for maintaining access to effective therapy during periods of high allergen exposure. They should not, however, be interpreted as proof that every treatment gap causes loss of protection or requires rescue corticosteroid therapy. The clinical complexity associated with multiple relevant allergies and prolonged or overlapping pollen seasons therefore supports individualized treatment planning rather than a uniform bottle-size recommendation. Limitations include the absence of direct RCT comparisons between BAK-preserved and preservative-free olopatadine, the lack of adherence studies specifically evaluating bottle size, heterogeneity between CAC and environmental studies, and variability in drop yield and labelled in-use periods. Future research should address these gaps prospectively, including same-molecule preserved versus preservative-free comparisons and studies evaluating adherence, cost, wastage, microbiological safety, and clinical outcomes across BID and once-daily regimens.

## 8. Conclusions

Olopatadine ophthalmic solution is a well-established topical antihistamine for SAC, with proven efficacy and a favorable safety and tolerability profile. Its clinically relevant pharmacological targets are accessible at the conjunctival surface, and current evidence does not establish that BAK-mediated enhancement of intraocular penetration is required for therapeutic efficacy; nevertheless, BAK remains an effective antimicrobial preservative in conventional multidose bottles. Preservative-free formulations may reduce preservative exposure, particularly during prolonged or repeated therapy, but direct evidence demonstrating superior outcomes for preservative-free versus BAK-preserved olopatadine is lacking. For the 0.1% BID regimen, larger fill volumes may reduce dispensing frequency when the validated in-use shelf life permits extended use, but an adherence benefit has not been demonstrated and once-daily formulations may offer comparable supply advantages with smaller volumes. Thus, preservative status and bottle size should be considered as part of a broader, individualized formulation choice that also includes dosing frequency, container microbiological safety, in-use shelf life, cost, wastage, ocular-surface status, and expected duration of allergen exposure.

## Figures and Tables

**Table 1:** Comparative efficacy of olopatadine ophthalmic solution versus other topical antihistamines in allergic conjunctivitis: summary of key evidence from randomized controlled trials and meta-analyses.

Comparator	Study/Source	Study Design	Primary Outcome (Ocular Itch)	Key Result	Favors
Ketotifen 0.025%	Cochrane review (Castillo et al., 2015) [10]	Meta-analysis (4 RCTs, n = 182)	Ocular itch at 14 days (0–3 scale)	MD −0.32 (95% CI: −0.59 to −0.06; *p* = 0.02); I^2^ = 83%	Olopatadine
Ketotifen 0.025%	Avunduk et al. (2005) [29]	RCT, 30 days, n = 39	Ocular itch (0–3 scale)	No significant difference between groups	Comparable
Ketotifen 0.025%	Kam et al. (2017) [24]	Systematic review & meta-analysis (5 RCTs)	Ocular itch	Pooled MD −0.22 (95% CI: −0.64 to 0.20; *p* = 0.31)	Comparable
Epinastine 0.05%	Fukushima & Ebihara (2014) [30]	RCT (CAC model), n = 50	Ocular itch at 7 min	MD −0.14 (*p* = 0.046); conjunctival hyperemia at 20 min: MD −0.23 (*p* = 0.027)	Olopatadine
Epinastine 0.05%	Kam et al. (2017) [24]	Meta-analysis (5 RCTs)	Ocular itch	Pooled MD −0.23 (95% CI: −0.50 to 0.03; *p* = 0.08)	Trend favoring olopatadine (NS)
Alcaftadine 0.25%	Fu et al. (2024) [31]	Meta-analysis (3 RCTs, n = 690 eyes)	Ocular itch	Pooled MD +0.39 (95% CI: 0.28–0.50; *p* < 0.00001; I^2^ = 0%)	Alcaftadine (CAC model)
Alcaftadine 0.25%	Fu et al. (2024) [31]	Meta-analysis	Ocular itch	Alcaftadine 0.25% superior to olopatadine 0.2% in CAC-based studies, particularly at earliest post-challenge time points	Alcaftadine (CAC model)
Alcaftadine 0.25%	Dudeja et al. (2019) [32]; Ayyappanavar et al. (2021) [33]	RCTs, environmental setting	TOSS/symptom scores	Comparable efficacy over 1–2 weeks	Comparable (real-world)
Cromolyn sodium 2%	Katelaris et al. (2002) [15]	RCT, 6 weeks, n = 185, multicenter (6 European countries + Australia), environmental setting	Ocular itch and redness (investigator-rated, 5-point and 9-point scales)	Olopatadine superior to cromolyn from day 14 onwards (itching and redness, both *p* < 0.05). Regression slopes for itching and redness 5× lower for olopatadine vs. cromolyn as pollen counts rose (*p* = 0.002 and *p* = 0.016). Olopatadine superior on Physicians’ Impression Scale on days 30 and 42 (*p* < 0.05)	Olopatadine
Cromolyn sodium 2%/Levocabastine 0.05%	Ciprandi et al. (2004) [16]	Post hoc analysis of 2 RCTs, 6 weeks, children aged 4–11 years (n = 52), environmental setting (grass pollen season)	Ocular itch and conjunctival redness (self-rated and slit-lamp)	Olopatadine significantly superior to cromolyn in itching (*p* = 0.010), redness (*p* = 0.003), and eyelid swelling (*p* = 0.034) during peak pollen period. Superior to levocabastine in redness (*p* = 0.040) and Physicians’ Impression Scale (*p* = 0.026). Well tolerated in all children	Olopatadine
All comparators	Krungkraipetch et al. (2025) [1]	Network meta-analysis (28 RCTs)	SUCRA ranking	Olopatadine 0.1%: SUCRA 0.88 (SAC), 0.85 (PAC)—highest ranked	Olopatadine

CAC, conjunctival allergen challenge; CI, confidence interval; MD, mean difference; NS, not statistically significant; PAC, perennial allergic conjunctivitis; RCT, randomized controlled trial; SAC, seasonal allergic conjunctivitis; SUCRA, Surface Under the Cumulative Ranking Curve; TOSS, Total Ocular Symptom Score.

**Table 2:** Major allergenic pollen taxa in temperate Europe: seasonal duration and illustrative implications for topical antihistamine drug supply [18,19].

Pollen Taxon	Representative Species	Typical Pollen Season (Temperate Europe)	Peak Season Duration	Illustrative Symptomatic Exposure Window (Weeks)	Estimated 5-mL Bottles (BID)	Estimated 10-mL Bottles (BID)
Betulaceae (birch, alder, hazel)	*Betula pendula*, *Alnus glutinosa*, *Corylus avellana*	January–May	4–6 weeks	4–6	1–2	1
Cupressaceae (cypress, juniper)	*Cupressus sempervirens*, *Juniperus* spp.	January–April	3–5 weeks	3–5	1–2	1
Oleaceae (olive, ash)	*Olea europaea*, *Fraxinus excelsior*	March–June	3–5 weeks	3–5	1–2	1
Platanaceae (plane tree)	*Platanus* × *hispanica*	March–April	2–3 weeks	2–3	1	1
Poaceae (grasses)	*Lolium*, *Dactylis*, *Phleum*, *Poa*	April–September	6–10 weeks	6–10	2–3	1–2
Ambrosia (ragweed)	*Ambrosia artemisiifolia*	July–October	4–8 weeks	4–8	1–2	1–2
Parietaria	*Parietaria judaica*, *P. officinalis*	March–October (Mediterranean)	8–12 weeks	8–12	2–3	1–2
Polyallergic patients (multiple clinically relevant taxa)	—	Variable; may span sequential pollen seasons	Individualized	Individualized	Not prespecified	Not prespecified

Note: BID, twice daily (one drop per eye per instillation, 4 drops/day total). The exposure windows shown are illustrative estimates derived from reported pollen-season patterns and do not constitute recommendations for continuous pharmacological treatment. Actual treatment duration should be individualized according to clinically relevant exposure and symptoms. Bottle requirements are theoretical arithmetic estimates based on approximately 100–120 drops per 5 mL bottle and 200–240 drops per 10 mL bottle. Actual drop yield varies by product and dispensing system, and safe use is additionally limited by the product-specific labelled in-use shelf life. These calculations should not be interpreted as prescribing recommendations or as evidence of improved adherence, clinical outcomes, or cost-effectiveness.

## Data Availability

No new data were generated or analyzed in this narrative review. All sources discussed are cited in the reference list.
